# Canopy Roughness: A New Phenotypic Trait to Estimate Aboveground Biomass from Unmanned Aerial System

**DOI:** 10.34133/2020/6735967

**Published:** 2020-12-08

**Authors:** Monica Herrero-Huerta, Alexander Bucksch, Eetu Puttonen, Katy M. Rainey

**Affiliations:** ^1^Department of Agronomy, Purdue University, West Lafayette, IN, USA; ^2^Department of Cartographic and Land Engineering, Higher Polytechnic School of Avila, University of Salamanca, Avila, Spain; ^3^Institute for Plant Sciences, College of Agriculture, Purdue University, West Lafayette, IN, USA; ^4^Department of Plant Biology, University of Georgia, Athens, GA, USA; ^5^Warnell School of Forestry and Natural Resources, University of Georgia, Athens, GA, USA; ^6^Institute of Bioinformatics, University of Georgia, Athens, GA, USA; ^7^Finnish Geospatial Research Institute, National Land Survey of Finland, Masala, Finland

## Abstract

Cost-effective phenotyping methods are urgently needed to advance crop genetics in order to meet the food, fuel, and fiber demands of the coming decades. Concretely, characterizing plot level traits in fields is of particular interest. Recent developments in high-resolution imaging sensors for UAS (unmanned aerial systems) focused on collecting detailed phenotypic measurements are a potential solution. We introduce canopy roughness as a new plant plot-level trait. We tested its usability with soybean by optical data collected from UAS to estimate biomass. We validate canopy roughness on a panel of 108 soybean [Glycine max (L.) Merr.] recombinant inbred lines in a multienvironment trial during the R2 growth stage. A senseFly eBee UAS platform obtained aerial images with a senseFly S.O.D.A. compact digital camera. Using a structure from motion (SfM) technique, we reconstructed 3D point clouds of the soybean experiment. A novel pipeline for feature extraction was developed to compute canopy roughness from point clouds. We used regression analysis to correlate canopy roughness with field-measured aboveground biomass (AGB) with a leave-one-out cross-validation. Overall, our models achieved a coefficient of determination (*R*^2^) greater than 0.5 in all trials. Moreover, we found that canopy roughness has the ability to discern AGB variations among different genotypes. Our test trials demonstrate the potential of canopy roughness as a reliable trait for high-throughput phenotyping to estimate AGB. As such, canopy roughness provides practical information to breeders in order to select phenotypes on the basis of UAS data.

## 1. Introduction

Solar radiation directly impacts crop growth by influencing biophysical parameters such as canopy photosynthetic rate, crop evapotranspiration, crop radiation capture, and water-use efficiency [1]. The underlying hypothesis of our study states that the morphological traits of the plant canopy are associated with the canopy interaction with solar radiation.

Remote sensing has the capability to measure canopy traits nondestructively in early growing stages, improving data quality over manual trait measurements and reducing time and cost for phenotyping [2, 3]. Over the last decade, unmanned aerial sensing (UAS) became a highlighted tool in plant phenotyping [4]. The easy control and operation of UAS in combination with improved accuracy, high temporal, radiometric, and spatial resolution of the data acquired, plus the possibility to fly them when soil conditions make fields inaccessible has led to a growing user community. Yet, this community needs automatic pipelines to fully exploit the potential of UAS-collected data.

The next breakthrough in breeding efficiency is expected to be highly dependent on automating the phenotyping process to link the genotype to the phenotype using genomic and phenotypic information throughout plant development [5]. For example, the characterization of quantitative traits from agricultural plant populations at the plot level allows complex phenotypes, such as yield, to be identified from RGB or hyperspectral imagery [6, 7]. These plant traits at the plot level recognized by images can be used to detect genetic markers and improve selection of highly efficient phenotypes. Therefore, developing automatic data processing pipelines to obtain phenotypic traits with new technologies like UAS platforms directly addresses the phenotypic bottleneck.

Aboveground biomass (AGB) of crops indicates the physiological conditions of the plant, affecting management decisions regarding crop productivity, fertilizer application, and pest control, as well as being a critical variable for plant phenotyping [5]. AGB is demarcated as a complex and multidimensional plant trait [8]. Recent studies reveal AGB's high correlation with point cloud-derived canopy volume metrics [8–11]. Active light detection and ranging (LiDAR) sensors have the capacity to penetrate and acquire 3D measurements of the crop, allowing plant parameter estimations [12–15]. However, the cost and weight of LiDAR sensors still remain a disadvantage to overcome. In contrast, photogrammetric passive sensors are lighter and less expensive. Therefore, UAS equipped with photogrammetric sensors are a cost-effective solution to collect plant canopy traits across a wide wavelength range with high spatial resolution [16–24]. In addition, structure from motion (SfM) offers the possibility to obtain 3D point clouds on the basis of 2D images taken from various viewpoints [25–27]. First, the view of each image is automatically determined, and subsequently, 3D coordinates are computed to get a dense and scaled point cloud of the scene [28].

Our study introduces canopy roughness as a new plot level trait that can be efficiently computed from UAS imaging data for large numbers of plots. The presented pipeline was tested in a multienvironmental soybean trial in Indiana (USA), with high spatial resolution data coming from UAS-based RGB imagery and field-measured AGB as ground truth. As a result, we present canopy roughness as an indicator for AGB that allows the selection of high performing phenotypes in large-scale plant breeding operations.

## 2. Materials and Methods

**Figure 1:**
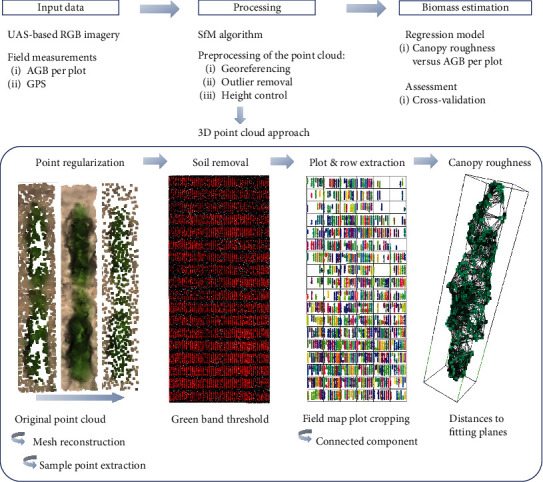
Schematic overview of the developed pipeline to estimate AGB by the proposed canopy roughness trait by 3D point clouds coming from RGB imagery. Canopy roughness by plot is calculated by a chain process once the point cloud of the study area is reached: (a) regularizing the point quantity of the field point cloud, (b) soil removal trough a green band filter, (c) plot and, subsequently, row extraction from the point cloud, and (d) computation of the canopy roughness using distances from points to best fitting plane.

The schematic overview of the pipeline visualizes data of RGB images and field measurements (Figure 1). The 3D point clouds were generated with the SfM method. Using the 3D point clouds as input, we compute the canopy roughness index per plot. In the statistical evaluation, we estimate biomass from trend lines resulting from regression models and assess estimates for robustness with a leave-one-out cross-validation.

### 2.1. Experimental Setup

Experiments were carried out at two locations in Indiana (USA) in 2018. Location 1 was at ACRE (the Agronomy Center for Research and Education from Purdue University) (40°28′20.5^″^N 86°59′32.3^″^W) and Location 2 was at Romney (40°14′59.1^″^N 86°52′49.4^″^W), about 27 km north from Location 1. 108 recombinant inbred lines from 32 families were planted. The panel includes lines from three classes of families: 16 from elite parents, 12 with diverse pedigrees, and four that are high-yielding under drought conditions [29].

The soil of the soybean fields was a silt loam with a pH of approx. 6.5. Both trials were planted at 2.5 cm depth in rows 0.76 m apart to a density of 35 seeds/m^2^ on May 22^nd^ for Location 1 and on May 17^th^ for Location 2. Each individual plot had 8 rows for a total of 108 plots. For the analysis, we eliminated the border plants such that only 6 rows per plot were analyzed. No fertilizers or herbicides for weed control were applied. Absence of water stress and adequate nutritional status in natural conditions during the growing season was monitored by measuring the water balance following the FAO 56 guide and soil analysis in the ACRE laboratory at Purdue University [29].

**Figure 2:**
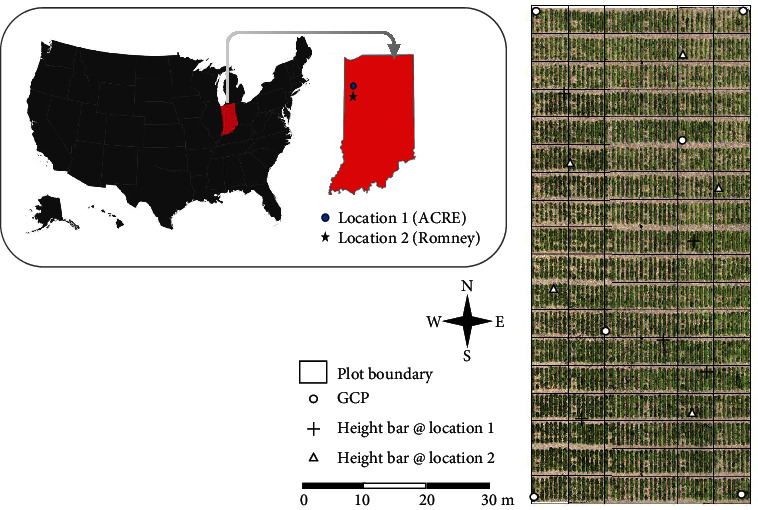
Test site locations in Indiana (left) and the setup of the soybean experiment with marked locations of each plot, height bars, and GCPs (right). GCPs are used to properly georeference the point cloud, and height bars are used to adjust and check the altitude accuracy.

Six GCPs (Ground Control Points) were placed on the ground for correct scaling and georeferencing in both trials. In addition, five height-fixed bars of equal length were randomly placed over the study area to control for height accuracy. All accuracy targets had high reflection markers for easy detection in the UAS images.  Figure 2 shows the test site location on the left and the experimental design on the right as an orthomosaic computed from the UAS data.

### 2.2. Data Acquisition

Data was collected for the early phenological growth stage R2, in which rows are visually distinguishable in the aerial images. The R2 stage is characterized by an open flower at one of the two uppermost nodes on the main stem with a completely developed leaf.

First, we did a topographic survey using the accuracy targets in the study area (6 GCPs and 5 height-fixed bars) with a Topcon GNSS device (Topcon corporation, Tokyo, Japan) using Real-Time Kinematic [30] for georeferencing. The eBee platform (senseFly, Lausanne, Switzerland) to collect imaging data is a fixed-wing UAS with on-board GPS, IMU, and magnetometer. The weight of the eBee is 700 g, and it carries a payload of 150 g. The on-board digital camera is controlled by the autopilot function during the flight. The senseFly S.O.D.A. (senseFly, Lausanne, Switzerland) was used as the photogrammetric sensor and has a focal length of 10.6 mm, a pixel size of 3 *μ*m, and a sensor size of 116.2 mm^2^ and produces images of 5742 × 3648 pixels.

Flight routes were planned and designed with the senseFly software (senseFly, Lausanne, Switzerland). The software calculates the flight strips, the camera orientation, and the image acquisition parameters for the autonomous flying mode. The photogrammetric flight configuration was set up with an along- and across-track overlap of 75%. The senseFly software estimated a flight altitude of 95 m for the required ground sample distance (2.54 cm). A total of 189 images for Location 1 and 142 images for Location 2 were obtained as an input to compute the 3D point cloud. The exposure time was fixed at 1/1000 sec with ISO (the International Organization of Standardization) 125 for both flights. The UAS collected imaging data at 43 DAP (day after planting) (July 4^th^) for Location 1 and at 46 DAP (July 2^nd^) for Location 2. The acquisition time for both flight campaigns was noon, due to the sun lighting conditions, minimizing the shadows captured by nadir images.

We collected AGB samples per plot at 48 DAP (July 9^th^) at Location 1 and 50 DAP (July 6^th^) at Location 2, by cutting all the stems at a 1-meter distance in each of two neighboring rows, roughly 2 cm above the ground. These samples were processed in a drying oven at 60.0°C until the weights stabilized; they were then weighed and analyzed by plot.  Table 1 represents the AGB data per plot reached in these conditions (Locations 1 and 2).

### 2.3. Processing of Aerial Data

UAS images were processed with the Pix4Dmapper software package (Pix4D SA, Lausanne, Switzerland) to georeference aerial images. The software output also includes the camera calibration, image orientation, and dense point cloud extraction. The software employs the GCPs' measurements to retrieve the camera's interior parameters and corrects for any systematic error or block deformation. As a result, point clouds are accurately georeferenced to the earth reference system World Geodetic System 84, specifying the error in this process.

From our experience, the automatically generated point clouds are likely to contain outlier points. Therefore, we implemented an outlier removal routine implemented in C++ using Point Cloud Library (PCL) [31], compiled and run on the Ubuntu 14.04 64-bit operating system, taking a 20-point neighborhood of each point into account. We remove all points that are more than 3 times the standard deviation away from the mean of the Gaussian distribution of all pairwise distances in the neighborhood [32]. Secondly, the distance to the underlying surface is analyzed by locally fitting a plane to the 20-point neighborhood. Again, the threshold to remove a point is set to 3 times the standard deviation from the mean of the Gaussian distribution of the point distances to the fitted plane. After completing the two outlier removal procedures, we identify the five height-fixed bars randomly placed within the point cloud. These bars were measured by the GNSS device, to easily locate them and in order to adjust the measured height to the *z*-coordinate (elevation) of the bars manually extracted from the point cloud.

#### 2.3.1. Regularizing the 3D Point Cloud

3D reconstruction from images typically generates point cloud datasets of varying point densities with frequent holes. The explication is that the accuracy of the depth map per image is dependent on the density and distribution of feature points used to align the images. To solve this issue, we regularize the point density in order not to influence the shape of the canopy surface used in the next calculations. To do that, we compute a mesh from the point cloud with the 3D Delaunay triangulation algorithm [33]. Once the mesh is computed, meshing gaps are repaired using planar triangulation [34] followed by smoothing of the mesh using a Laplacian filter to a 10 cm radius around user selected locations [35]. Next, randomly sampled points over the mesh are extracted by fixing a desired density (500 points/m^2^) and obtaining a restored point cloud. We then apply Dart Throwing Poisson Disk sampling to the point cloud to make the points appear more uniform by culling those points that are close to a randomly selected point [36]. In this step, a threshold based on Euclidean distance between points of 1 cm is set. After this process, a significant reduction of points is achieved because the Poisson subsampling approach considers the local point distribution. This method retains key elements of the structure, preserving a good amount of detail while significantly reducing the number of points, particularly on the ground plane. Thus, the regularized point cloud is achieved.

#### 2.3.2. Soil Removal from the 3D Point Cloud

We implemented a radiometric classification to separate the vegetation from the ground that exploits the information contained in the visible spectrum of the UAS images. In doing so, each point of the point cloud is labeled with the average color from the pixels of all images contributing to the reconstruction of the point during the SfM process considering the viewing angle. To analyze the colored point cloud, we set a cut-off in the green band of 115 in an 8-bit scale to automatically classify vegetation and soil, tested by a visual inspection. The cut-off is derived from the physical characteristics of the sensor configuration, the light conditions, the crop type, and the phenological state of the vegetation.

#### 2.3.3. Plot and Row Segmentation

We used rapidlasso LAStools [37] to crop out each plot from the point cloud, specifically the tool named “lasclip” using the SHP file already generated based on the field map. Once the plots are extracted, the rows within the plot are defined by the connected component labeling algorithm. For that, the space is divided into a regular 3D grid of 0.15 m step. Thus, every single point is inside this grid. Labels are assigned based on their grid neighborhood connectivity. Clusters formed by few points are removed (50 points in this case), considered as they cannot represent a homogeneous growth of the soybean plants. Therefore, the method to extract individual rows consists of a discretization of the point cloud through a 3D grid.

#### 2.3.4. Canopy Roughness

We introduce canopy roughness as a new trait for crop phenotyping. Canopy roughness is a numerical value which characterizes the irregularities of the canopy surface measured by high resolution 3D point clouds coming from RGB imagery acquired by UAS. We compute canopy roughness for each preprocessed plot in two steps:

(i) We estimate the *point roughness* as the Euclidean distance between each point and the best fitting plane of the neighbors circumscribed by a sphere of a user defined radius (0.10 m in this case). The roughness per point is measured in meters
(ii) We calculate *canopy roughness* at the plot level from the interquartile range and the median of the point roughnesses of all points (Equation (1)).
  (1) $$\begin{array}{c} \text{CR}={\text{IQR}}^{\text{med}}, \end{array}$$
  where CR is the canopy roughness at plot level, med is the median, and IQR is the interquartile range of the roughness values from all the points within the plot. Canopy roughness is measured in m^2^ and represents the distribution of point roughness

Equation (1) is derived from the best empiric correlation with AGB.

### 2.4. Biomass Estimation

Regression models that correlate CR and AGB on the plot level were analyzed. A leave-one-out cross-validation (LOOCV) was chosen to evaluate the model estimation strength to minimize potential overfitting and allow for accurate and unbiased assessment [38]. To accurately and robustly analyze the regression model, several statistical metrics were calculated, in particular, the coefficient of determination (*R*^2^) and the *p* value. In addition, the root mean square error (RMSE), the relative RMSE (RRMSE), the average systematic error (ASE), and the mean percent standard error (MPSE) were calculated. These metrics were computed as follow:

(2) $$\begin{array}{c} \text{RMSE}=\sqrt{\frac{\sum_{i=1}^{n}{\left(x_{r}^{i}-x_{\text{AGB}}^{i}\right)}^{2}}{n}}, \end{array}$$

(3) $$\begin{array}{c} \text{RRMSE}=100*\frac{\text{RMSE}}{{\bar{x}}_{\text{AGB}}}, \end{array}$$

(4) $$\begin{array}{c} \text{ASE}=\frac{100}{n}*\overset{n}{\underset{i=1}{\sum }}\frac{x_{r}^{i}-x_{\text{AGB}}^{i}}{x_{A\text{GB}}^{i}}, \end{array}$$

(5) $$\begin{array}{c} \text{MPSE}=\frac{100}{n}*\overset{n}{\underset{i=1}{\sum }}\left|\frac{x_{r}^{i}-x_{\text{AGB}}^{i}}{x_{\text{AGB}}^{i}}\right|, \end{array}$$

where *x*_*r*_^*i*^ is the canopy roughness of the *i*^th^ plot, *x*_AGB_^*i*^ is the measured AGB within the *i*^th^ plot, ${\bar{x}}_{\text{AGB}}$ is the mean of the measured AGB per plots, and *n* is the number of plots in the testing dataset.

## 3. Experimental Results

Canopy roughness was computed for the two trial locations. The 3D point clouds of the field experiments were obtained with a georeferencing mean RMSE error of 1.1 cm (Location 1) and 1.7 cm (Location 2), using the GNSS measurements of the six GCPs per location. Our processing pipeline achieved a spatial resolution of more than 700 points per m^2^ for Location 1 and more than 1,100 points per m^2^ for Location 2. This notable difference in the number of points between the two locations is due to the Pix4D settings for the dense point cloud extraction step, optimal point density for Location 1 and high point density for Location 2. After performing outlier removal, the point clouds contained 2,177,801 points (Location 1) and 3,501,126 points (Location 2). Next, the elevation of the point cloud was adjusted using the *z*-coordinate of the five reference height bars measured by GNSS. The adjustment resulted in a height error of less than 2.11 cm and 1.97 cm for both locations, respectively.

**Figure 3:**
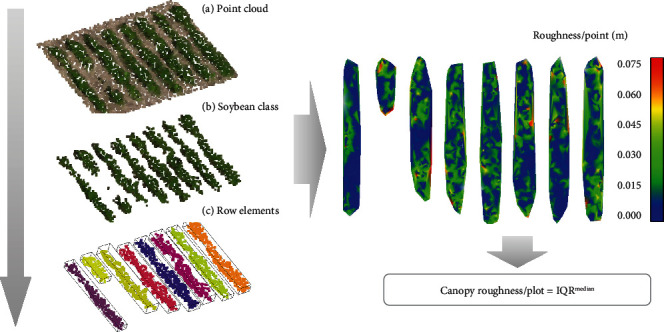
Point cloud processing steps for a random plot: (a) plot extraction from the point cloud of the study area, (b) filtering of the point cloud to obtain the soybean points, and (c) individual row extraction by connected component algorithm and removal of less than 50-point clusters (left); computation of the individual roughness of all the points to finally calculate the canopy roughness per plot by statistic parameters from the individual roughness distribution (right).

We first regularize the point cloud density by computing and repairing the 3D mesh from the original point cloud. The regularized point cloud is obtained by sampling points over the mesh, setting a value of 500 points/m^2^. This value is selected as a compromise between the computational cost and the irregularities on the crop surface. Now, by the Dart Throwing Poisson Disk method, we sample the point cloud by setting a minimum Euclidean distance between points of 1 cm. This value gives us enough detail of the crop surface. In the next stage, the point cloud is classified into vegetation and ground using a threshold on the green band of a value of 115 in an 8-bit scale. To evaluate the performance of the final classification, 1,000 independent points were randomly selected and manually checked. The overall accuracy of the classification was higher than 99.68%, and the Kappa coefficient was higher than 0.996 for both locations. These validation metrics indicate that incorrectly classified points have insignificant influence on the estimated trait. 243,608 points were classified as soybean plants (including flower points) from a total of 791,572 points (30.8%) for Location 1 and 323.859 from a total of 962.317 points (33.6%) for Location 2. After the classification, plots and rows were extracted using a 3D grid step less than 0.150 m. Components with less than 50 points were omitted. Next, we computed point roughness as the distance to the best fitting plane within a sphere radius of 0.10 m for all soybean points within each row (the extreme rows from each plot are eliminated in order to avoid border effects). Following, canopy roughness per plot is computed according to Equation (1).  Figure 3 illustrates the point cloud processing results of each step of the pipeline on an example plot.

**Figure 4:**
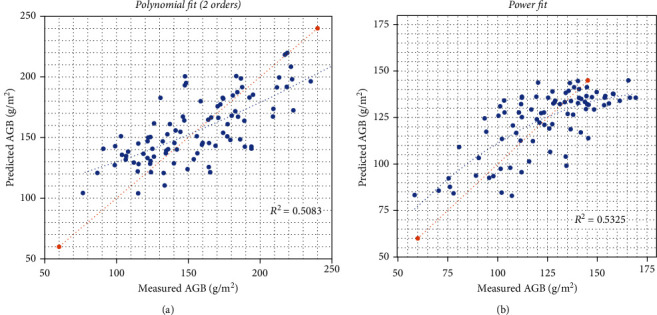
Cross-validation scatter plots for field-measured AGB versus estimated AGB at Location 1 (a) and Location 2 (b) using the best regression model. 1 : 1 line marked as a red discontinuous line and regression line as a blue discontinuous line.

After computing canopy roughness per plot, univariate regression models to estimate AGB from canopy roughness at a plot level were derived. The error metrics associated with the regression analysis at a significance level of 0.05 for a two-tailed Gaussian distribution are shown in Table 2. A total of 10 and 17 outliers from plot-level canopy roughness were detected for Locations 1 and 2, respectively, reaching a maximum standard residual of -1.98 and 1.99 at each location. The best model from each location is compared with corresponding measured values using a 1 : 1 scatterplot (Figure 4), ending up with a *p* value < 0.001 for both locations.

The LOOCV method excluded one sample per trial to determine the model and used all other samples for computing the model in both trials. Table 2 summarizes the error metrics (Equations (2)–(5)) as averages of 106 plots for Location 1 and 103 plots for Location 2 where the plants are grown properly, from which the best model per site was selected.

**Figure 5:**
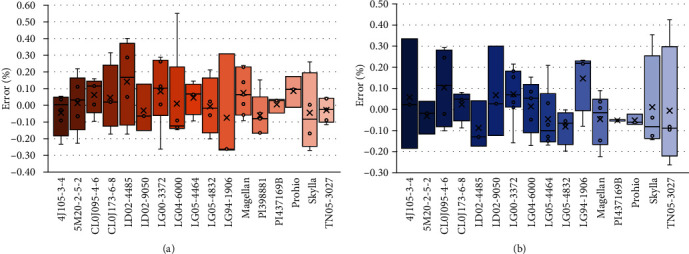
Errors (%) for AGB estimation grouped by genotype at Location 1 (a) and Location 2 (b).

The robustness of the proposed methodology for AGB estimation was evaluated by soybean genotype.  Figure 5 and Table 3 present an analysis of the genotype dependency of our AGB estimations. Error metrics were grouped by genotypes. With these results, we affirm that the best biomass prediction was found for genotype PI437169B. The weakest predictive strength for biomass was found for genotype LG94-1906. The best site-independent prediction was made for genotype LG04-6000, which showed consistent results at both experimental sites. This means that genotype LG04-6000 shows overall less influence of gene∗environment interaction in our model. Therefore, the proposed new trait (canopy roughness) confirms efficacy to characterize AGB variations among genotypes.

## 4. Discussion

The proposed data processing pipeline computes canopy roughness at the plot level from high resolution point cloud data. In our first application, we used soybeans as a demonstration model. Regression analysis revealed a coefficient of determination of over 0.5 with field-measured AGB for two different locations. Moreover, phenotypic correlation depends on genetic and environmental correlations [39]. This dependency might explain differences between the regression models used for AGB estimations for Locations 1 and 2, even if the planted genotypes are exactly the same at both experimental sites (Figure 4). Notably, the model performance is inverted between Locations 1 and 2; for example, linear, polynomial, and logarithmic models perform better in Location 1, but worse in Location 2. Moreover, Table 2 shows that CR and AGB are directly proportional in the linear model for Location 1 and inversely proportional for Location 2. Our data indicates that the environment acts in different directions at both trial sides, as well as the interaction between the genotype and the environment [40]. However, at least one more year of data collection with the new method is needed to formulate a sound hypothesis. When the model is computed with enough plots to consider the variability within the study field, the calibration procedure will neutralize these differences in the interactions. Therefore, the model capabilities could be improved with additional data and through the combination of different traits and sensors. Key techniques based on testing data, feature combination, and model selection currently depend on highly specialized knowledge from both computer science and plant science [41]. In addition, canopy roughness evidences the potentiality of distinguishing among genotypes required for high-throughput phenotyping. As we demonstrated, the estimation quality is genotype dependent; however, we acknowledge that more successive studies should be conducted, including a wide variety of genotypes with more replications. In that way, the statistical power to differentiate genotypes of extracted canopy roughness could be improved.

The results presented in this research paper demonstrate the potential of canopy roughness as a new trait closely related to AGB in soybeans. The canopy roughness is a simple but efficient metric for plant scientists, convenient for automated high-throughput crop biomass phenotyping. Specifically, this study evaluates the capability of 3D modeling, combined with regression models to effectively give accurate predictions of AGB in soybean experimental fields. Moreover, this study highlights the power of UAS platforms as a rapid, accurate, and cost-effective tool in collecting high spatial resolution optical data for high-throughput phenotyping.

One advantage to consider is that the proposed pipeline does not require a reference flight over the experimental field, making an easy flight campaign, planning, and data processing. In addition, the registration errors that occur when a point cloud of flights from different time points is registered to each other are nonexistent. As a possible limitation, we found that the model has to be computed by each trial due to different environmental and genotype characteristics. However, a standardized test plot per genotype is enough to efficiently compute the model by environment. In the future, our approach can be effectively applied to other plant species and UAS platforms for high-throughput phenotyping and even with LiDAR-collected point clouds. Still, more comprehensive studies are required on diverse crop species at different phenological stages to calibrate the algorithm parameters to other plants or even to monitor other aspects as crop health. Additionally, future work should explore the suitability of canopy roughness as a trait to high-throughput AGB phenotyping using a large replicability and number of soybean genotypes. Another technical aspect to investigate is the use of voxel-based methods that structure point clouds to regularize point density [42]. We argue that point cloud regularization can be optimized in a way that the morphology of individual plants becomes computable from UAS data.

Altogether, our study introduced canopy roughness as a new trait to high-throughput phenotyping for UAS platforms. We believe that plant breeders can make immediate use of canopy roughness as a new trait to improve phenotyping selection in ongoing trials. We also envision future applications of canopy roughness in precision agriculture if combined with automated pest management and on-line yield estimates.

## Figures and Tables

**Table 1:** Statistics of AGB field measurements per plot: location (site), date of AGB measurements (DAP), number of plots where AGB was collected (# plots), mean AGB (g/m^2^) (mean), median AGB (g/m^2^) (median), standard deviation based on the mean (g/m^2^) (std), minimum AGB (g/m^2^) (min), maximum AGB (g/m^2^) (max), kurtosis coefficient (kurtosis), and skewness coefficient (skewness).

Site	DAP	# plots	Mean	Median	std	Min	Max	Kurtosis	Skewness
1	48	106	150.30	148.10	42.31	10.45	251.62	0.52	-0.21
2	50	103	124.62	126.29	35.09	35.92	230.92	0.90	0.19

**Table 2:** Validation statistics of univariate regression models for AGB estimation (significance at 0.05 level (2-tailed)) by canopy roughness at the two locations (the best model per experimental test site is highlighted in boldface).

Site	Model		*R* ^2^	RMSE (g/m^2^)	RRMSE (%)	ASE (%)	MPSE (%)
1	Linear	*y* = 3187.2*x* − 2938.3	0.506	26.273	16.74	3.19	14.73
	Power	*y* = 274.22*x*^20.061^	0.488	26.468	16.87	1.58	14.37
	Exponential	*y* = 3*E* − 07*E*^20.622x^	0.487	26.853	17.11	-0.26	14.15
	**Polynomial**	**y** = 2764**x**^2^ − 2189**x** − 324.27	**0.506**	**26.272**	**16.74**	**3.15**	**14.72**
	Logarithmic	*y* = 3099.3ln(*x*) + 247.71	0.506	26.274	16.74	3.13	14.72

2	Linear	*y* = −1705.5*x* + 1751.2	0.502	16.706	13.28	2.08	11.86
	**Power**	**y** = 60.331**x**^−14.87^	**0.531**	**16.736**	**13.31**	**1.04**	**11.84**
	Exponential	*y* = 3*E* + 08*e*^−15.5*x*^	0.531	19.129	15.21	-5.65	12.62
	Polynomial	*y* = 7886.9*x*^2^ − 16844*x* + 9114.6	0.503	16.691	13.27	2.12	11.90
	Logarithmic	*y* = −1637ln(*x*) + 46.94	0.502	16.703	13.28	2.05	11.86

*R* ^2^: coefficient of determination; RMSE: root mean square error; RRMSE: relative RMSE; ASE: average systematic error; MPSE: mean percent standard error.

**Table 3:** Validation statistics for AGB estimation grouped by genotype, with 3 or more estimations per experimental site (extreme values per experimental test site are highlighted in boldface).

Location	Genotype	No. of plots	RMSE	RRMSE	ASE	MPSE
1 (ACRE)	4J105-3-4	4	19.75	13.63	-4.34	0.70
	5M20-2-5-2	5	27.19	16.38	1.32	0.65
	CL0J095-4-6	5	15.66	10.32	7.84	2.40
	CL0J173-6-8	4	28.16	16.25	4.50	0.43
	LD02-4485	4	34.05	24.13	14.04	7.12
	LD02-9050	3	19.18	11.34	-2.99	2.15
	LG00-3372	6	25.12	18.22	8.29	4.38
	LG04-6000	5	30.63	18.94	1.10	11.00
	LG05-4464	4	16.01	10.19	4.63	1.61
	LG05-4832	4	23.69	14.28	-0.66	1.46
	**LG94-1906**	**3**	**49.67**	**27.83**	**-7.41**	**10.31**
	Magellan	8	17.13	11.38	6.33	0.26
	PI398881	5	23.23	13.84	-6.24	3.31
	**PI437169B**	**3**	**6.70**	**3.98**	**0.55**	**1.18**
	Prohio	3	17.13	11.10	8.56	0.42
	Skylla	4	35.06	20.49	-4.42	6.50
	TN05-3027	5	11.64	7.33	-2.91	0.52

2 (Romney)	4J105-3-4	3	25.32	19.08	5.80	0.77
	5M20-2-5-2	3	10.70	8.10	-3.13	3.86
	CL0J095-4-6	4	20.26	17.00	10.46	6.16
	CL0J173-6-8	4	7.95	7.33	2.34	1.16
	LD02-4485	3	18.24	13.07	-8.69	4.31
	LD02-9050	3	21.54	15.98	6.82	10.00
	LG00-3372	7	14.15	12.89	7.25	2.60
	LG04-6000	5	11.42	9.82	1.46	3.42
	LG05-4464	6	19.82	13.66	-4.59	2.13
	LG05-4832	4	17.87	12.41	-8.19	0.04
	**LG94-1906**	**4**	**19.53**	**19.64**	**14.81**	**1.98**
	Magellan	6	14.87	12.23	-4.56	3.71
	**PI437169B**	**3**	**7.08**	**5.16**	**-5.11**	**1.58**
	Prohio	3	7.71	5.46	-5.08	0.72
	Skylla	4	19.52	15.92	1.27	3.11
	TN05-3027	4	23.32	19.58	-0.34	6.55

## Data Availability

The datasets used and analyzed during the current study are available from the corresponding author on reasonable request.
